# Comparison of tibial fracture plate length, placement, and fibular integrity effects on plate integrity through finite element analysis

**DOI:** 10.1038/s41598-024-64990-w

**Published:** 2024-06-24

**Authors:** Byung Hoon Lee, Yeokyung Kang, Sung Ha Cho, Myung Moon, Jae Ang Sim, Jungsung Kim

**Affiliations:** 1 Department of Orthopaedic Surgery, Gachon University College of Medicine, 21, Namdong-Daero 774 Beon-Gil, Namdong-Gu, Incheon, Republic of Korea; 2 Central Research and Development Center, Corentec Company Ltd., 33-2, Banpo-Daero 20-Gil, Seocho-Gu, Seoul, Republic of Korea; 3 Gachon University College of Medicine, Incheon, Republic of Korea

**Keywords:** Anatomy, Engineering, Biomedical engineering

## Abstract

Minimally invasive plate osteosynthesis is the most commonly used minimally invasive surgery technique for tibial fractures, possibly involving single or dual plate methods. Herein, we performed a finite element analysis to investigate plate strength according to the plate type, length, and presence of a fibula by constructing a three-dimensional tibia model. A thickness of 20 mm was cut 50 mm distal from the lateral plateau, and the ligaments were created. Plates were modeled with lengths of 150, 200, and 250 mm and mounted to the tibia. Screws were arranged to avoid overlapping in the dual plating. The von-Mises stress applied to the plates was measured by applying a load of 1 body weight. Dual plates showed the least stress with low displacement, followed by medial and lateral plates. As the plate length increased, the average stress gradually decreased, increasing plate safety. The difference in the influence of the fibula depending on the presence of proximal fibula osteotomy showed that the average stress increased by 35% following proximal fibula osteotomy in the D1(Plate type: Dual plate, Medial plate length: 150 mm, Lateral plate length: 200 mm, Non Proximal fibula osteotomy) and D1P(Plate type: Dual plate, Medial plate length: 150 mm, Lateral plate length: 200 mm, Proximal fibula osteotomy) models, confirming the necessity of the fibula model. There is no consensus guideline for treatment of this kind of fracture case. A single fracture plate can decrease the risk of skin damage, ligament damage, and wound infection, but because of its design, it cannot provide sufficient stability and satisfactory reduction of the condylar fragment, especially in cases of comminution or coronal fracture. So, these results will help clinicians make an informed choice on which plate to use in patients with tibial fractures.

## Introduction

Tibial fractures are a common condition caused by sudden trauma. These fractures can be painful, require prolonged recovery, and lead to complications^1,2^. Every year, 492,000 tibia, fibula, and ankle fractures occur in the United States^3^. Of these, 77,000 lead to prolonged hospitalization, and 825,000 patients are treated at a hospital yearly^4^. Among them, tibia plateau fractures account for 1% of all fractures^5^.

Tibial fractures require medical treatment, and treatment options may vary depending on the type of injury, severity, and complications involved^6,7^. In particular, plate fixation has been shown to be an effective option when fractures reach metaphysis^8,9^. In this technique, single plating fixes the medial and lateral plates according to the direction, while dual plating simultaneously fixes the medial and lateral plates. In 2015, a study by Yao et al. compared lateral plates used in single and dual plating in 86 patients and reported no significant difference in patient satisfaction between the two groups^10^. A study by Neogi et al. in 2015 also compared the 2-year follow-up of a group of 29 patients with lateral plates and a group of 32 with dual plates, finding no significant difference in the knee scores between the two groups^11^. On the other hand, a study by Çağlar et al. in 2021 showed that dual plates yielded a better range of motion values than lateral plates^12^.

A single plate operation is simple because the soft tissue in the medial direction of the tibia is thin, reducing the surgical time^13^. However, the lateral plate is the gold standard as it has a high strength to convert the tensile force into a compressive force^14,15^. A previous study, in which finite element analysis (FEA) was conducted, reported that medial plates experience higher stress^16^. In contrast, the strength of the medial plate was reported to be higher in a cadaver study comparing the mechanical stability of the medial and lateral plates^17^.

In this study, the strength of the dual, medial, and lateral plates was compared, and FEA was used to examine the changes in strength with increases in the length of the plates. Fracture of the tibial metaphysis was assumed through three-dimensional (3D) modeling based on image data of an actual patient, and the plates were 3D modeled at lengths of 150–250 mm to fit the patient's bones. We hypothesis that as the plate length increased, the stress will decrease and the fibula integrity or not will impact the stress increasing. Also, the hypothesis that plate displacements can impact differently on two condition mentioned in prior was established.

## Materials and methods

### Bone model construction using patient image data

**Figure 1:**
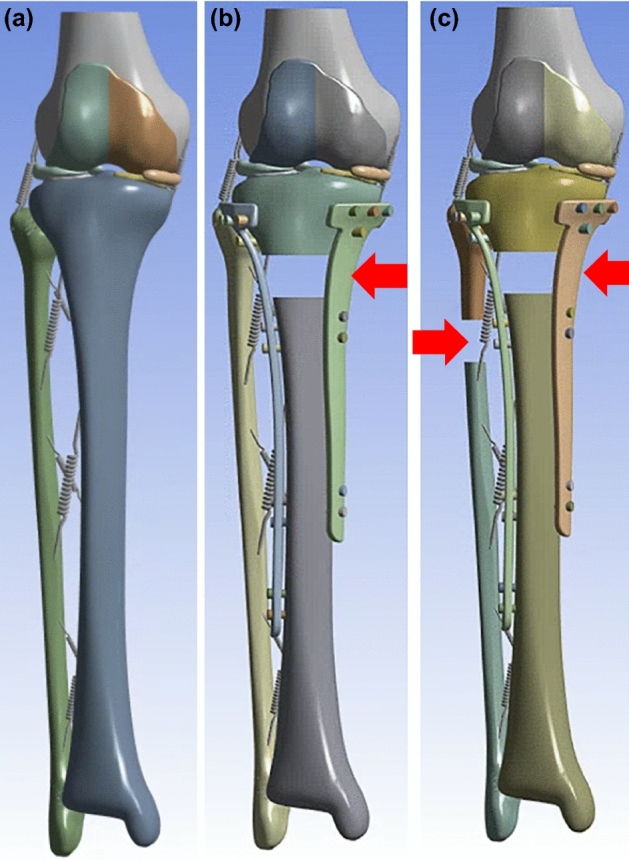
The tibia fracture plate model with and without PFO. (**a**) The intact bone model (**b**) The tibia fracture plate model without PFO (indicated by red arrow) (**c**) The tibia fracture plate model with PFO (indicated by red arrows). PFO, proximal fibula osteotomy.

The bone model used in this study was designed based on imaging data of the intact right lower extremity, which side doesn’t have pathological problems, of a female individual belonging to the Republic of Korea aged 45 years with a height of 152.8 cm and a weight of 73.4 kg. Computed tomography (CT) data of the entire right lower extremity and magnetic resonance imaging (MRI) data of the knee joint were used. The model was reconstructed using imaging data in Digital Imaging and Communications in Medicine format and Mimics 16.0 (Materialize NV, Belgium) software. The 3D model information of the distal femur, tibia, and fibula was collected from the CT data. The 3D models of the distal femur, proximal tibia, femur cartilage, tibia cartilage, and meniscus were obtained from the MRI data. The reconstructed 3D models were transferred to 3-Matic 8.0 (Materialize NV, Belgium) software and combined in alignment with their respective positions using the global registration function. The distal femur and proximal tibia obtained from the MRI data were moved and aligned based on the tibia and femur bones, and the cartilages and menisci of the MRI data were moved with the model aligned based on the bones. As shown in Fig. 1a, a bone intact model constructed using the distal femur, tibia, and fibula from the CT data and cartilage and meniscus from the MRI data was used in this study.

A thickness of 20 mm was cut 50 mm distal from the lateral plateau of the constructed bone intact model to create a tibial fracture, as shown in Fig. 1b. In addition, a bone model with a 20-mm osteotomy located 80 mm distal from the lateral plateau was created to confirm the influence of the presence of the fibula, as shown in Fig. 1c on which proximal fibula osteotomy (PFO) was performed^18^.

### Establishment of the ligaments

**Figure 2:**
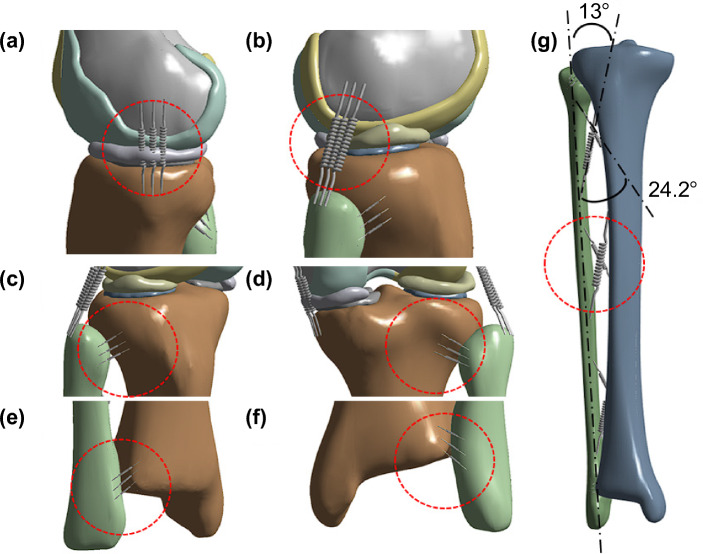
The location of all ligaments. (**a**) The MCL (b) The LCL (**c**) The proximal anterior tibiofibular ligament (**d**) The proximal posterior tibiofibular ligament (**e**) The distal anterior tibiofibular ligament (**f**) The distal posterior tibiofibular ligament (**g**) The interosseous membrane. All these ligaments are marked by round dotted circles. MCL, medial collateral ligament; LCL, lateral collateral ligament.

Ligaments were constructed in the form of a spring in the bone model to make the motion of the bone model more realistic and to strengthen the constraints of the interpretation between the femur and tibia and between the tibia and fibula (Fig. 2). The locations of the ligaments were specified based on video data and existing research^19,20^, and the ligaments were fixed using three springs for each type of ligament. The medial collateral ligament and lateral collateral ligament were constructed as shown in Fig. 2a,b, and the tibiofibular ligament was constructed in the anterior and posterior directions on the proximal and distal regions, as shown in Fig. 2c–f. The interosseous membrane was divided into two types and was fixed to the anterior and posterior directions, as shown in Fig. 2g, while the anterior interosseous membrane was fixed at a 13° angle and the posterior interosseous membrane at a 24.2° angle based on the fibula axis^21^.

### Plate model construction

A tibia plate was modeled and applied to the previously constructed tibial fracture bone model using 3-Matic 8.0 (Materialize NV, Belgium) software. The plate was modeled with a thickness of 4.2 mm according to the shape of the tibia in the medial and lateral directions and was mounted on the tibia at a distance of 2 mm from the tibia. The plate was modeled with lengths of 150, 200, and 250 mm, and a lateral plate with a longer length than a medial plate was used for the dual plating according to the general surgical procedure. The plate models applied according to the interpretation models are shown in Supplementary Table S1.

The space between the tibia and plate was fixed by modeling a cylindrical screw with a diameter of 4 mm; the screws of the medial and lateral plates were positioned perpendicular to the tibia but inserted in deviating directions to avoid overlapping. The proximal tibia was fixed with four screws, and the shaft was fixed with two; two screws were further added for each 50-mm increase in plate length. All screws were fixed by penetrating the cortical bone on the opposite side.

**Figure 3:**
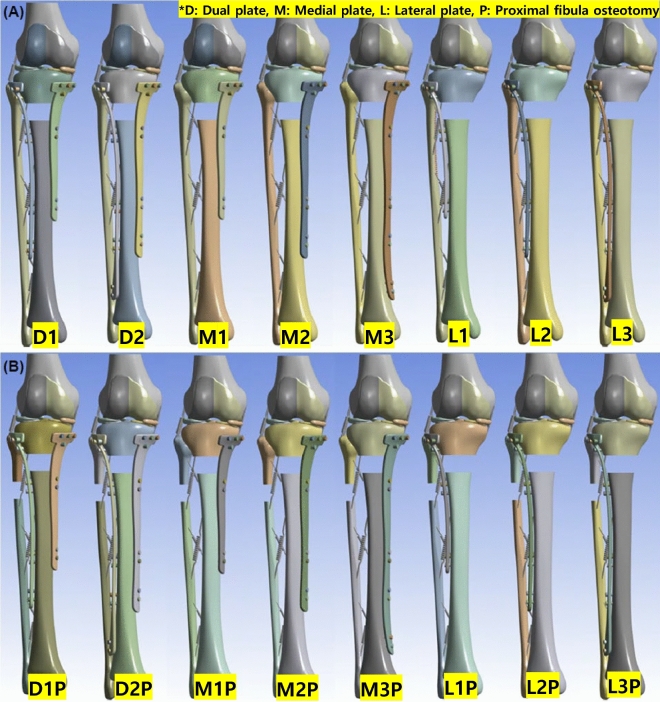
The tibia fracture plate model with and without PFO. (**a**) The tibia fracture plate model without PFO (**b**) The tibia fracture plate model with PFO. PFO, proximal fibula osteotomy.

The 16 interpretive models were constructed as shown in Fig. 3. Remeshing was performed using Ansys 2022R2 (Ansys, Inc., US) software, and each model's number of elements and nodes are presented in Supplementary Table S2.

### Material properties

The properties of the materials applied in the current study are shown in Supplementary Table S3^22–27^. The anisotropy properties were used for the material properties of the cortical bone, and Young's modulus of 1061 MPa and Poisson's ratio of 0.225 were used for the cancellous bone. Material properties of Young's modulus of 12 MPa and Poisson's ratio of 0.45 were applied for the cartilages and Young's modulus of 80 MPa and Poisson's ratio of 0.3 for the menisci. For ligaments, the stiffness of a single spring was applied to each site. In addition, the models were designed such that the ligaments were not loaded during compression but took the load of stiffness under tension.

### Verification of the bone model using a finite element analysis

The convergence of a finite element model was performed using the error rate based on the interpretation of the mesh factor number. We aimed to evaluate the accuracy of worst-case specifications and standard model efficacy using the “convergence” function with an “adaptive mesh refinement” approach that involved repeated interpretations with increasing mesh factors in the Ansys software program. In the repeated interpretation process, we used the ratio of factors that slowly increased with the “refinement depth 1/2” setting and an acceptable percentage of chance for convergence set at 5% to evaluate each finite element model’s errors^28^.

### Load and boundary conditions

The load and boundary conditions were set using Ansys 2022R2 (Ansys, Inc., US) software. The contact conditions are shown in Supplementary Table S4. The tibia and fibula were wholly fixed on the side contacting the talus in the distal direction, and the cortical and cancellous models of the bone were also fully fixed. Complete fixations were also made between the femur and cartilage, as well as along the tibia, cartilage, and meniscus. A friction coefficient of 0.2 was applied between the femur cartilage and meniscus and between the femur cartilage and tibia cartilage, where the knee joint moves with friction. The coefficient of friction of the contact between the tibia cortical bone and fibula cortical bone was 0, although it was given a state of no separation^29–31^. Furthermore, the spaces between the plate and screws and between the screws and bone were fixed entirely to prevent any micromotion^32–34^.

A load of 1 body weight (720 N) was applied to a proximal metaphysis where the femur was cut^29^ and to the medial and lateral condyle at a ratio of 2:1 (480 N Medial: 240 N Lateral).

### Region of interest (ROI)

**Figure 4:**
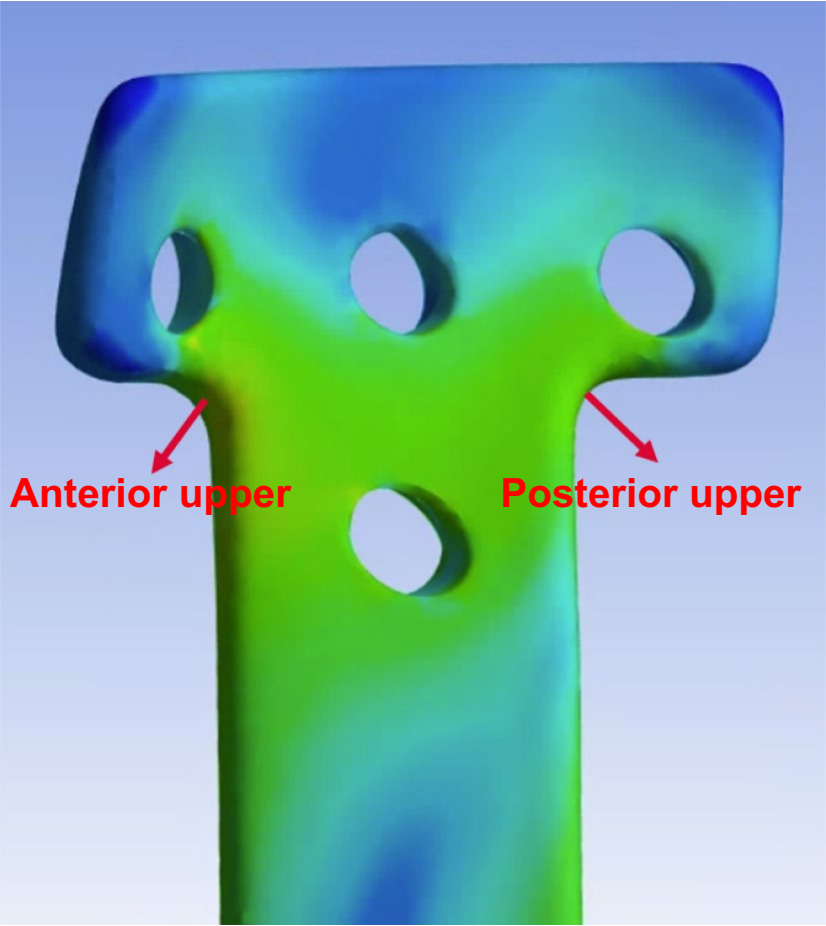
The upper neck of the ROI. ROI, region of interest.

The results of peak von-Mises stress, average von-Mises stress, peak displacement, and average displacement of the entire plate were derived. The comparison was performed by measuring the anterior direction of the upper part of the plate and the posterior direction of the neck, which are the ROIs in Fig. 4 most commonly measured in existing plate studies^35^.

### Ethics approval

Ethics approval was exempted from Gachon University Gil Medical Center IRB due to the retrospective design and anonymized data. And all research was performed in accordance with relevant guidelines and regulations, and include that informed consent was obtained from participant and their legal guardians.

## Results

### Tibial fracture plate model without PFO

**Figure 5:**
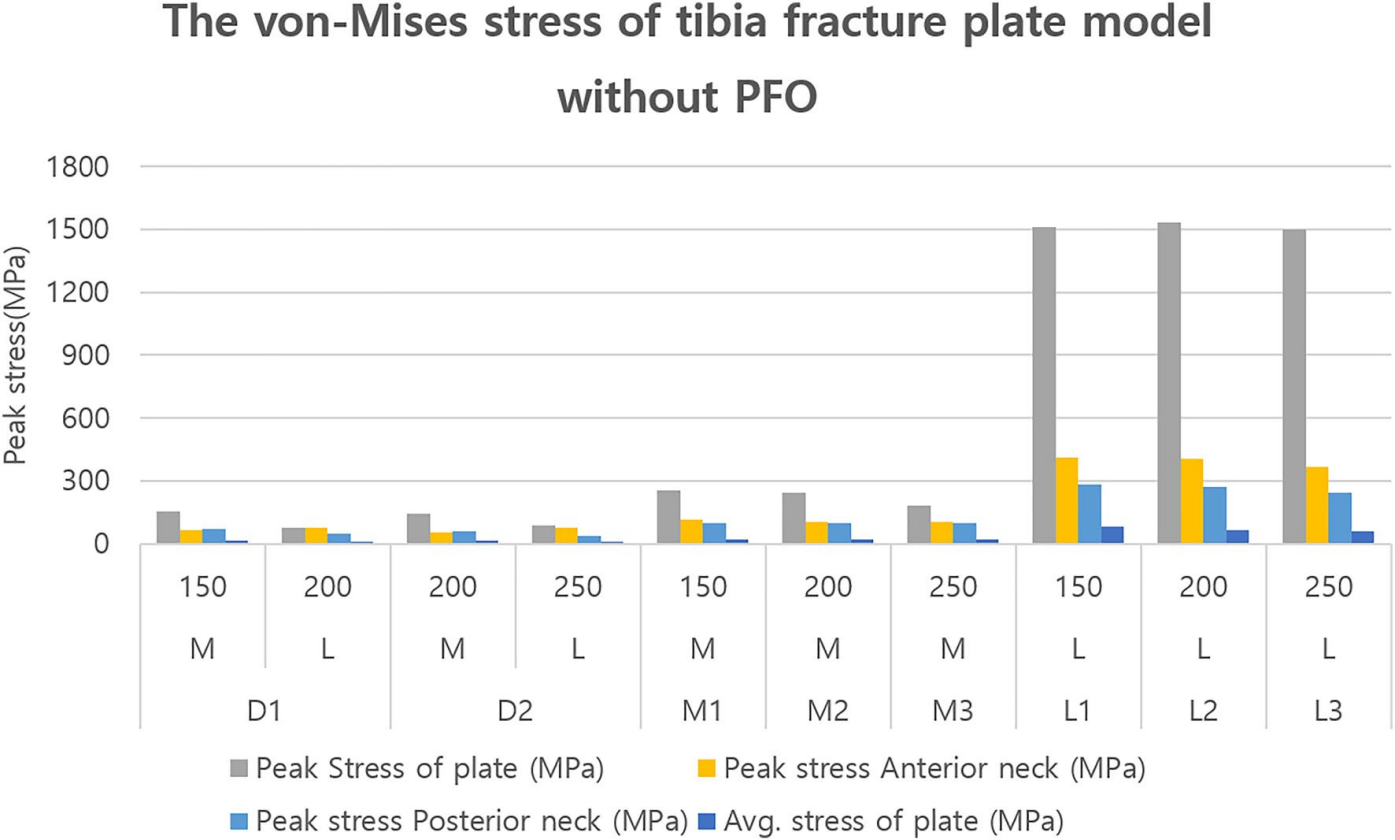
Stresses calculated for the tibia fracture plate model without PFO. PFO, proximal fibula osteotomy.

**Figure 6:**
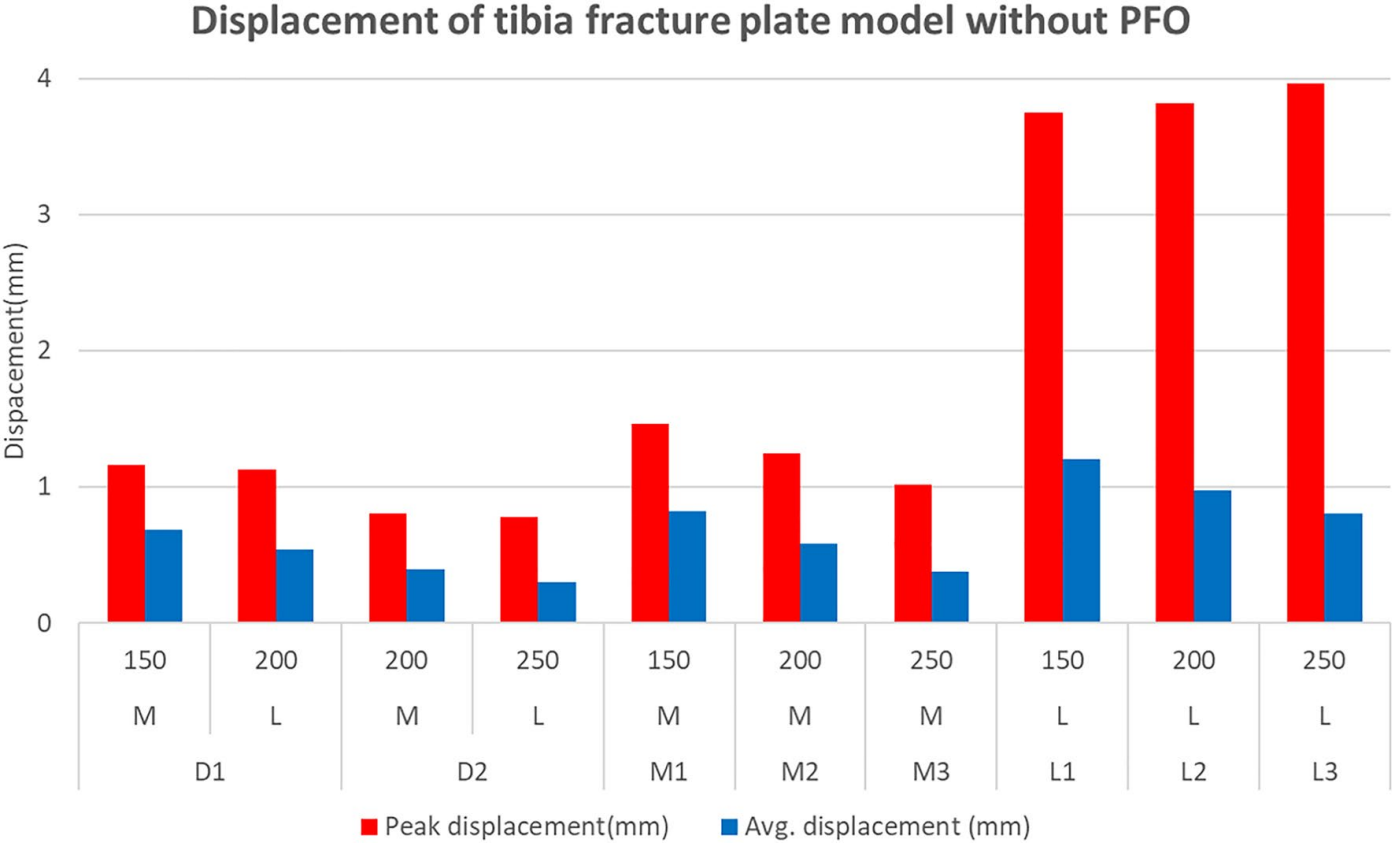
Displacements of the tibia fracture plate model without PFO. PFO, proximal fibula osteotomy.

Supplementary Tables S5, S6 and Figs. 5 and 6 show the results of the FEA of the tibial fracture plate model, in which the influence of the fibula was present as no PFO was performed. The results showed that the peak stress of the plate was 154.64 MPa for the medial plate and 75.14 MPa for the lateral plate of the D1 model, with corresponding values of 145.63 MPa and 87.55 MPa for the D2 model, respectively. The medial plates of the M1, M2, and M3 models yielded peak stresses of 256.91, 241.46, and 181.66 MPa, respectively, while those of the lateral plates of the L1, L2, and L3 models were 1510.6, 1531.4, and 1499.4 MPa, respectively.

The average stress of the plate, the second comparison variable, was derived as 14.06 MPa for the medial plate and 9.56 MPa for the lateral plate of the D1 model, with corresponding values of 12.56 and 8.68 MPa for the D2 model, respectively. The medial plates of the M1, M2, and M3 models yielded maximum stresses of 20.03, 17.95, and 17.51 MPa, respectively, and the lateral plates of the L1, L2, and L3 models yielded maximum stresses of 80.31, 65.02, and 59.50 MPa, respectively.

Third, the maximum stress in the anterior direction of the upper plate was 62.46 MPa for the medial plate and 75.14 MPa for the lateral plate of the D1 model, with corresponding values of 51.00 MPa and 73.53 MPa for the D2 model, respectively. The maximum stress of the medial plates of the M1, M2, and M3 models were 113.06, 105.26, and 101.18 MPa, respectively, and the maximum stress of the lateral plates of the L1, L2, and L3 models were 413.64, 403.74, and 366.98 MPa, respectively.

In addition, the maximum stress in the posterior direction of the upper plate was 68.48 MPa for the medial plate and 45.90 MPa for the lateral plate of the D1 model, with corresponding values of 61.38 MPa and 37.57 MPa for the D2 model, respectively. The medial plates of the M1, M2, and M3 models yielded maximum stresses of 100.08, 99.92, and 96.96 MPa, respectively, and the lateral plates of the L1, L2, and L3 models yielded values of 281.45, 273.07, and 243.95 MPa, respectively.

The peak displacement of the medial plate was 1.16 mm, and that of the lateral plate was 1.13 mm in the D1 model, with corresponding values of 0.808 mm and 0.782 mm for the D2 model, respectively. The medial plates of the M1, M2, and M3 models yielded values of 1.46, 1.25, and 1.02 mm, respectively, while the lateral plates of the L1, L2, and L3 models yielded values of 3.75, 3.82, and 3.96 mm, respectively.

The average displacement of the plate was 0.682 mm for the medial plate and 0.536 mm for the lateral plate of the D1 model, with corresponding values of 0.392 mm and 0.298 mm for the D2 model, respectively. The medial plates of the M1, M2, and M3 models yielded values of 0.822, 0.585, and 0.376 mm, respectively, while the lateral plates of the L1, L2, and L3 models yielded values of 1.204, 0.976, and 0.801 mm, respectively.

### Tibial fracture plate model with PFO

**Figure 7:**
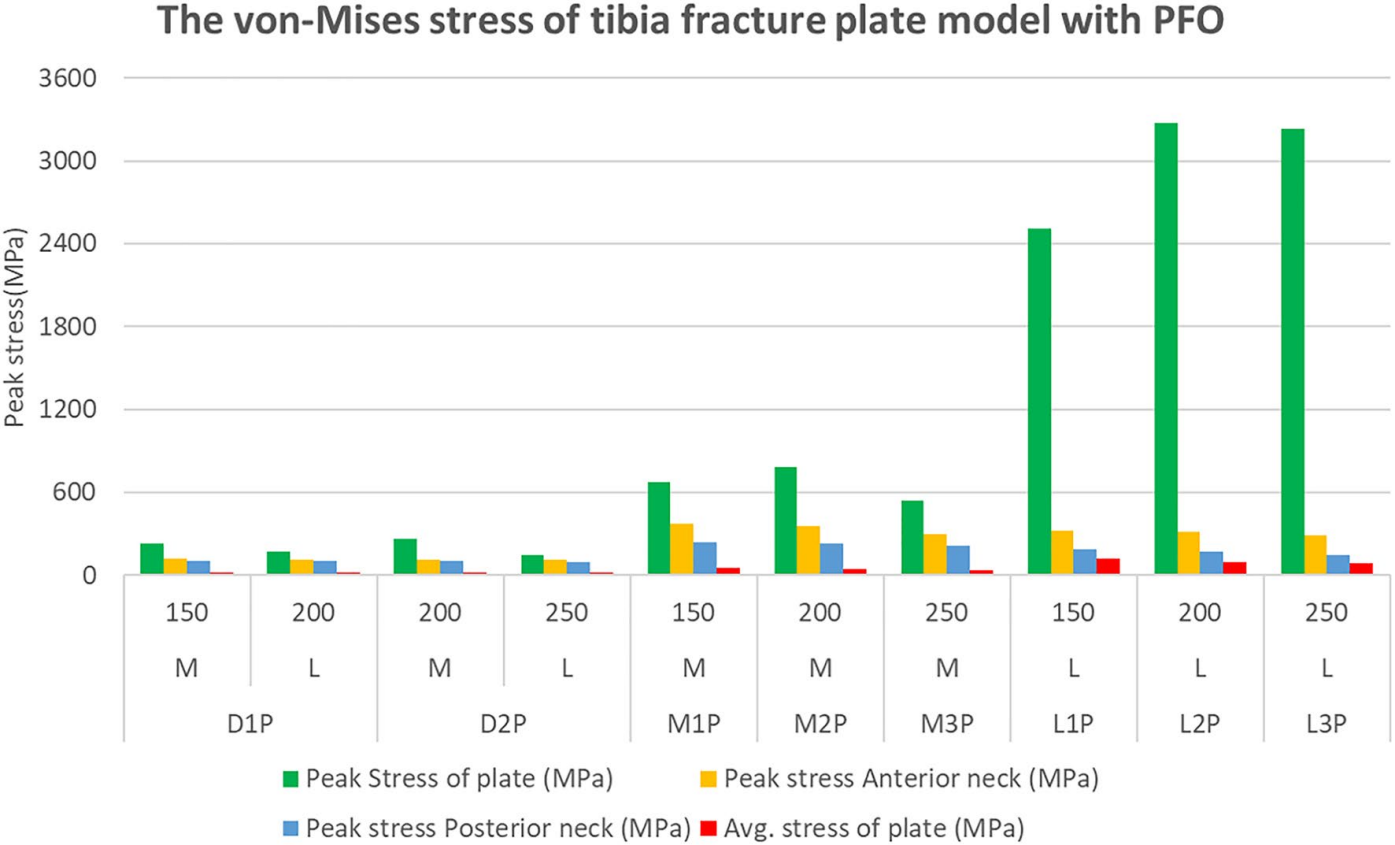
Stresses calculated for the tibia fracture plate model with PFO. PFO, proximal fibula osteotomy.

**Figure 8:**
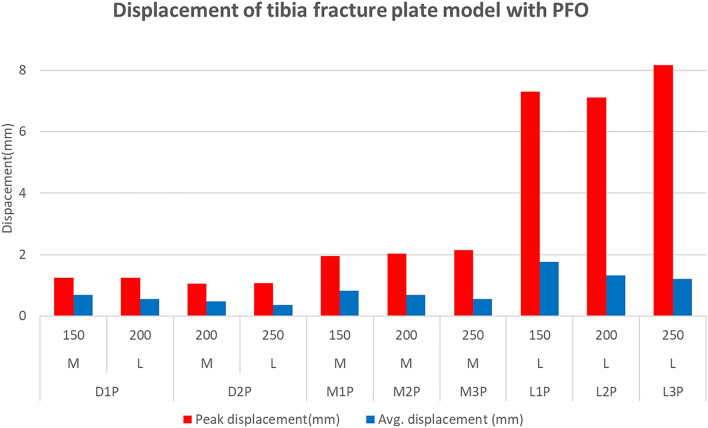
Displacements calculated for the tibia fracture plate model with PFO. PFO, proximal fibula osteotomy.

The FEA results of the tibial fracture plate model not influenced by the fibula due to the PFO are shown in Supplementary Tables S7, S8 and Figs. 7, 8. The peak stress of the plate, the first variable for comparison, was 227.19 MPa for the medial plate and 173.37 MPa for the lateral plate of the D1P model, with corresponding values of 257.8 MPa and 145.6 MPa for the D2P model, respectively. The medial plates of the M1P, M2P, and M3P models yielded values of 675.39, 781.34, and 538.48 MPa, respectively, while the lateral plates of the L1P, L2P, and L3P models yielded values of 2512.2, 3274.6, and 3235.3 MPa, respectively. The total stress distributions of the eight models are shown in Fig. 7.

The average stress of the plate, the second variable for comparison, was 18.98 MPa for the medial plate and 14.94 MPa for the lateral plate of the D1P model, with corresponding values of 17.26 MPa and 14.47 MPa for the D2P model, respectively. The medial plates of the M1P, M2P, and M3P models yielded values of 49.82, 42.47, and 38.39 MPa, respectively, while those for the lateral plates in the L1P, L2P, and L3P models were 121.57, 93.59, and 88.42 MPa, respectively.

The maximum stress in the anterior direction of the upper plate was 116.01 MPa for the medial plate and 114.20 MPa for the lateral plate of the D1P model, with corresponding values of 113.62 MPa and 107.68 MPa for the D2P model, respectively. The medial plates of the M1P, M2P, and M3P models yielded values of 372.35, 352.52, and 293.73 MPa, respectively, and the lateral plates of the L1P, L2P, and L3P models yielded values of 320.83, 312.81, and 287.89 MPa, respectively.

In addition, the maximum stress of the upper plate in the posterior direction was 98.30 MPa for the medial plate and 98.21 MPa for the lateral plate of the D1P model, with corresponding values of 101.62 MPa and 96.82 MPa for the D2P model, respectively. The medial plates of the M1P, M2P, and M3P models yielded values of 234.94, 224.33, and 210.92 MPa, respectively, and the lateral plates of the L1P, L2P, and L3P models yielded values of 184.19, 167.04, and 144.99 MPa, respectively.

The peak displacement of the medial and lateral plates of the D1P model was identical at 1.24 mm, although the corresponding values were 1.06 mm and 1.07 mm for the medial and lateral plates of the D2P model, respectively. The medial plates of the M1P, M2P, and M3P models yielded values of 1.96, 2.04, and 2.15 mm, respectively, and the values for the lateral plates of the L1P, L2P, and L3P models were 7.31, 7.12, and 8.16 mm, respectively.

The average displacement of the plate was 0.685 mm for the medial plate and 0.546 mm for the lateral plate of the D1P model, with corresponding values of 0.474 mm and 0.372 mm for the D2P model, respectively. The medial plates of the M1P, M2P, and M3P models yielded values of 0.824, 0.683, and 0.558 mm, respectively, and the lateral plates of the L1P, L2P, and L3P models yielded values of 1.762, 1.325, and 1.204 mm, respectively.

## Discussion

The results according to the type and length of the plate were significant in the tibial fracture plate model, which was influenced by the fibula as PFO was not performed. Stress and displacement according to the type of attached plate were lowest for the dual plate, followed by the medial plate and then the lateral plate. However, the peak stress of the lateral plate did not show any tendency toward decreasing stress or displacement with the increase in length.

Tibial fracture plate models without the influence of fibula due to PFO showed the same tendency as those of tibial fracture plate models without PFO. The difference in the influence of the fibula depending on the presence of PFO was also significant. The peak stress at the upper plate neck was larger in models L1 through L3 than in models M1 through M3 but was smaller in models L1P through L3P than in models M1P through M3P.

Many previous clinical studies comparing conventional single and dual plates reported no significant difference in patient knee scores post-treatment^10,11^. However, in this study, we found that the average stress of the dual plate without PFO was 28% lower than that of the medial plate and 87% lower than that of the lateral plate, showing that the dual plate had superior mechanical strength. A similar study that measured the stress of the bone with a simple fracture reduced with single medial, single lateral, and dual plating found that the effect on bones did not differ significantly between plate types^36^. Given this, depending on the severity of the tibial fracture, operations involving dual plate fixation after incisions on both sides may have increased complexity and complications. Choosing a medial plate may therefore be an alternative^37^.

In 2012, Shon and Park performed plate osteosynthesis of distal tibial fractures and reported that medial and lateral plates showed good clinical results in treating tibial fractures^38^. In the current study, the average stress of the lateral plate was 301% higher than that on the medial plate, confirming that the medial plate had a much better strength. Considering the shorter surgical duration due to thin, soft tissue and based on the average stress results derived from this study, we believe that the medial plate has more advantages over the lateral plate^13^. However, further research is needed in overweight patients undergoing plate fixation using a medial plate because more stress may be applied to the plate^39^.

The stress decreased with the increase in plate length. The average and peak stress of the dual and medial plates decreased slightly with increasing plate length. The average stress of the lateral plate decreased with increasing plate length, although the peak stress was higher at 200 mm than at 150 mm. This result is similar to that of a 2019 study by Cao et al., in which the stress and displacement of the lateral plate did not decrease as length increased^16^. The peak stress of the L2 and L2P models did not decrease as the length increased, and the corresponding value was larger than the basic properties of the material, therefore, this part could be considered fracture. However, in the trend according to the length and attachment direction of the plate that was reported in our study, the average stress decreased as the plate length of L2 and L2P increased, so it is a meaningful hypothesis that structural safety increases as the plate length increases. This is in accordance with previous published clinical research paper which decided that the use of relatively longer plates is a vital technical factor that can reduce the risk of plate fixation failure^40,41^.

This study showed that stress increased in all models when PFO was performed. The average stress increased by 42% in M1 compared with D1 and by 162% in M1P compared with D1P, and the difference in average stress between the dual and medial plates increased. In particular, the peak stress tendencies of the medial and lateral plates on the neck (the selected ROI) were altered. The peak stress of the neck in models M1 through M3 without PFO was lower than that of models L1 through L3, although the peak stress of the neck in models M1P through M3P, which was influenced by the fibula removed through PFO, was larger than that of models L1P through L3P. Previous studies have also shown that the fibula delivers 7.12–16.00% of the weight load^42,43^, and the change in stress tendencies after PFO in this study justifies that the fibula should be considered in tibia plate analysis. According to the above results, it can be confirmed that when PFO was performed and the plate was attached to the lateral side, the peak stress of the anterior and posterior neck of the plate was lower than when PFO was not performed. Additionally, it can be found that the stress is lower at shorter length plate of PFO with plate attached to the lateral side. This is clinically effective in cases where the patient's lateral plate must be attached in a situation where the patient's tibia and fibula fractures occur together unintentionally. Unlike other cases, it can be confirmed that a shorter plate produces lower stress and is clinically effective.

Compared to the stress value, the displacement value also showed a similar trend depending on the plate attachment position and length, and it was confirmed that the FEA where PFO was performed showed a larger displacement value. In addition, the fact that the lateral plate had a lower displacement than the medical plate in the dual plate (D1, D2) where PFO was not performed suggests that fibula influenced the support role of the lateral plate. And considering that displacement of more than 3.0 mm occurred in both L1-L3 and L1P-L3P, the plate in this case can be judged to be fractured, similar to the situation estimated from the stress value.

This study has several limitations. The most important limitation of this study is that the analysis was conducted with a bone model of a single case. There is thus a need to statistically examine the conclusions of the current study using more diverse bone models. Second, the bone model was constructed on the assumption that a bone fragment was removed due to a severe tibial fracture defect. New analysis results could be derived if it is assumed that the bone fragment was not lost. Third, a static simulation of weight loading was applied instead of a dynamic simulation that applies periodic loads on the knee joint. Therefore, it is possible that the derived displacement was lower than the actual value^44^. Finally, the bone model was modelled with cortical bone part except of bone marrow and spongy part. Regardless of later mentioned parts, cortical bone part is most high strength compared with other part, and when we start this study, we assumed same property all of the variables. In the same property condition, it doesn’t affect the FEA result whether there are bone mellow and spongy part. But, as this condition can causes difference of real clinical situation, we mention bone model construction as limitation in this paper.

## Conclusion

By examining the von-Mises stress by plate type, this study found the dual plate had superior mechanical strength, and for all plates, the average plate stress decreased with the increase in plate length. On the other hand, peak stress did not show the same tendency as average stress. Additionally, stress increased in all models when PFO was performed, and the difference in average stress between the dual and medial plates increased. The change in stress tendencies after PFO in this study suggests that the fibula should be considered in tibia plate analysis.

### Supplementary Information

Supplementary Information.

## Data Availability

The datasets used and/or analyzed during the current study available from the corresponding author on reasonable request.

## References

[1] Gunnar B. J. Andersson, Jeannette Bouchard, Kevin J. Bozic, *et al.* The Burden of Musculoskeletal Diseases in the United States. **2,** 148, *Bone and Joint Decade 2002-USA-2011*.

[2] Johnathan H, Petros Koutsogiannis; Alex Jahangir. Tibia Fractures Overview, *StatPearls (2023)* 30020639

[3] Praemer, A., Furner, S. & Rice, D.P. *Musculoskeletal Conditions in the United States*. 83–88 (American Academy of Orthopedic Surgeons, Rosemont, IL, 1999).

[4] Miller, N. C. & Askew, A. E. Tibia fractures: an overview of evaluation and treatment. *Orthop. Nurs.* **26**, 216–223 (2007). 17882096 10.1097/01.NOR.0000284648.52968.27

[5] Elsoe, R. *et al.* Population-based epidemiology of tibial plateau fractures. *Orthopedics.* **38**, e780–e786 (2015). 26375535 10.3928/01477447-20150902-55

[6] Bhandari, M., Guyatt, G. H., Swiontkowski, M. F. & Schemitsch, E. H. Treatment of open fractures of the shaft of the tibia. *J. Bone Joint Surg. Br.* **83**, 62–68 (2001). 11245540 10.1302/0301-620X.83B1.0830062

[7] Johnson, B. & Christie, V. Open tibial shaft fractures: a review of the literature. *Internet J. Orthop. Surg.* **9**, 1. 10.5580/1f16 (2007). 10.5580/1f16

[8] Huang, P. *et al.* A comparisive study between intramedullary interlocking nail and plate-screw fixation in the treatment of tibial shaft fractures. *Zhongguo Gu Shang.* **21**, 261–263 (2008). 19102183

[9] He, G. C., Wang, H. S., Wang, Q. F., Chen, Z. H. & Cai, X. H. Effect of minimally invasive percutaneous plates versus interlocking intramedullary nailing in tibial shaft treatment for fractures in adults: a meta-analysis. *Clinics (Sao Paulo).* **69**, 234–240 (2014). 24714830 10.6061/clinics/2014(04)03 PMC3971355

[10] Yao, Y. *et al.* A comparison of lateral fixation versus dual plating for simple bicondylar fractures. *Knee* **22**, 225–229 (2015). 25747747 10.1016/j.knee.2015.02.002

[11] Neogi, D. S., Trikha, V., Mishra, K. K., Bandekar, S. M. & Yadav, C. S. Comparative study of single lateral locked plating versus double plating in type C bicondylar tibial plateau fractures. *Indian J. Orthop.* **49**, 193–198 (2015). 26015609 10.4103/0019-5413.152478 PMC4436486

[12] Çağlar, C. *et al.* Comparative analysis of single lateral locked plate and double locked plate application in the treatment of bicondylar tibial plateau fractures. *Cureus* **13**, e19298. 10.7759/cureus.19298 (2021). 34877228 10.7759/cureus.19298 PMC8645974

[13] Anuar-Ramdhan, I. M. & Azahari, I. M. Minimally invasive plate osteosynthesis with conventional compression plate for diaphyseal tibia fracture. *Malays. Orthop. J.* **8**, 33–36 (2014). 26401234 10.5704/MOJ.1411.008 PMC4536398

[14] Hak, D.J. & Stewart, R.L. Tension band principle*. AO Principles of Fracture Management*, 2nd ed (AO Publishing, Davos, Switzerland, 2007).

[15] Rouhi, G. & Amani, M. A brief introduction into orthopaedic implants: screws, plates, and nails. (2012)

[16] Cao, Y., Zhang, Y., Huang, L. & Huang, X. The impact of plate length, fibula integrity and plate placement on tibial shaft fixation stability: a finite element study. *J. Orthop. Surg. Res.* **14**, 52 (2019). 30767784 10.1186/s13018-019-1088-y PMC6376681

[17] Ratcliff, J. R., Werner, F. W., Green, J. K. & Harley, B. J. Medial buttress versus lateral locked plating in a cadaver medial tibial plateau fracture model. *J. Orthop. Trauma.* **21**, 444–448 (2007). 17762474 10.1097/BOT.0b013e318126bb73

[18] Wang, X. *et al.* Proximal fibular osteotomy: a new surgery for pain relief and improvement of joint function in patients with knee osteoarthritis. *J. Int. Med. Res.* **45**, 282–289 (2017). 28222626 10.1177/0300060516676630 PMC5536585

[19] Kiapour, A. *et al.* Finite element model of the knee for investigation of injury mechanisms: development and validation. *J. Biomech. Eng.* **136**, 011002. 10.1115/1.4025692 (2014). 24763546 10.1115/1.4025692 PMC5101024

[20] Sun, J. *et al.* Finite element analysis of the valgus knee joint of an obese child. *Biomed. Eng. Online* **15**, 158. 10.1186/s12938-016-0253-3 (2016). 28155677 10.1186/s12938-016-0253-3 PMC5260062

[21] Elamrani, D., Aumar, A., Wavreille, G. & Fontaine, C. Comparative morphometry of the antebrachial and crural interosseous membranes: preliminary study for the use of the crural interosseous membrane in the surgical repair of the antebrachial interosseous membrane tears. *Surg. Radiol. Anat.* **36**, 333–339 (2014). 24036679 10.1007/s00276-013-1199-9

[22] Chitsazan, A., Rouhi, G., Abbasi, M., Pezehski, S. & Tavakoli, S. A. H. Assessment of stress distribution in ankle joint: simultaneous application of experimental and finite element methods. *Int. J. Exp. Comp. Biomech.* **3**, 45–61 (2015). 10.1504/IJECB.2015.067681

[23] Kim, S. H., Chang, S. H. & Jung, H. J. The finite element analysis of a fractured tibia applied by composite bone plates considering contact conditions and time-varying properties of curing tissues. *Compos. Struct.* **92**, 2109–2118 (2010). 10.1016/j.compstruct.2009.09.051

[24] Hopkins, A. R., New, A. M., Rodriguez-y-Baena, F. & Taylor, M. Finite element analysis of unicompartmental knee arthroplasty. *Med. Eng. Phys.* **32**, 14–21 (2010). 19897397 10.1016/j.medengphy.2009.10.002

[25] Cho, H. J. & Kwak, D. S. Mechanical properties and characteristics of the anterolateral and collateral ligaments of the knee. *Appl. Sci.* **10**, 6266. 10.3390/app10186266 (2020). 10.3390/app10186266

[26] Marchetti, D. C. *et al.* The proximal tibiofibular joint: a biomechanical analysis of the anterior and posterior ligamentous complexes. *Am. J. Sports Med.* **45**, 1888–1892 (2017). 28339288 10.1177/0363546517697288

[27] Beumer, A., van Hemert, W. L., Swierstra, B. A., Jasper, L. E. & Belkoff, S. M. A biomechanical evaluation of the tibiofibular and tibiotalar ligaments of the ankle. *Foot Ankle Int.* **24**, 426–429 (2003). 12801200 10.1177/107110070302400509

[28] Han, P. *et al.* Evaluation of biomechanical stability of newly developed revision total knee arthroplasty through strain and stress distribution analysis within the tibia: Finite element analysis. *J. Biomed. Eng. Res.* **34**, 14–23 (2013). 10.9718/JBER.2013.34.1.14

[29] Pan, D. *et al.* Effects of proximal fibular osteotomy on stress changes in mild knee osteoarthritis with varus deformity: a finite element analysis. *J. Orthop. Surg. Res.* **15**, 375 (2020). 32883311 10.1186/s13018-020-01894-1 PMC7469409

[30] Nie, Y. *et al.* Upper partial fibulectomy improves knee biomechanics and function and decreases knee pain of osteoarthritis: a pilot and biomechanical study. *J. Biomech.* **71**, 22–29 (2018). 29449003 10.1016/j.jbiomech.2017.12.004 PMC7426888

[31] Ünal, Ö. K., Dağtaş, M. Z., Demir, C., Najafov, T. & Ugutmen, E. The effects of proximal fibular osteotomy on the knee and ankle joints: a finite element analysis. *Acta Chir. Orthop. Traumatol. Cech.* **88**, 313–320 (2021). 34534062 10.55095/achot2021/047

[32] Cox, T., Kohn, M. W. & Impelluso, T. Computerized analysis of resorbable polymer plates and screws for the rigid fixation of mandibular angle fractures. *J. Oral Maxillofac. Surg.* **61**, 481–487 (2003). 12684967 10.1053/joms.2003.50094

[33] Atac, M. S., Erkmen, E., Yücel, E. & Kurt, A. Comparison of biomechanical behaviour of maxilla following Le Fort I osteotomy with 2- versus 4-plate fixation using 3D-FEA. Part 1: advancement surgery. *Int. J. Oral Maxillofac. Surg.* **37**, 1117–1124 (2008). 19027268 10.1016/j.ijom.2008.10.004

[34] Coquim, J. *et al.* Biomechanical analysis using FEA and experiments of metal plate and bone strut repair of a femur midshaft segmental defect. *Biomed. Res. Int.* **2018**, 4650308. 10.1155/2018/4650308 (2018). 30420962 10.1155/2018/4650308 PMC6211160

[35] Jang, Y. W. *et al.* Role of an anatomically contoured plate and metal block for balanced stability between the implant and lateral hinge in open-wedge high-tibial osteotomy. *Arch. Orthop. Trauma Surg.* **138**, 911–920 (2018). 29546620 10.1007/s00402-018-2918-9

[36] Forna, N. *et al.* Treatment of C1 1 (AO 41) tibial plateau fracture: a finite element analysis of single medial; lateral and dual plating. *Exp. Ther. Med.* **23**, 198. 10.3892/etm.2022.11121 (2022). 35126701 10.3892/etm.2022.11121 PMC8794547

[37] Shah, S. N. & Karunakar, M. A. Early wound complications after operative treatment of high energy tibial plateau fractures through two incisions. *Bull. NYU Hosp. Jt. Dis.* **65**, 115–119 (2007). 17581103

[38] Shon, O. J. & Park, C. H. Minimally invasive plate osteosynthesis of distal tibial fractures: a comparison of medial and lateral plating. *J. Orthop. Sci.* **17**, 562–566 (2012). 22570013 10.1007/s00776-012-0241-9

[39] Lee, A. K., Cooper, S. A. & Collinge, C. Bicondylar tibial plateau fractures: a critical analysis review. *JBJS Rev.* **6**, e4. 10.2106/JBJS.RVW.17.00050 (2018). 29461986 10.2106/JBJS.RVW.17.00050

[40] Beltran, M. J., Collinge, C. A. & Gardner, M. J. Stress modulation of fracture fixation implants. *JAAOS-J. Am. Acad. Orthop. Surg.* **24**(10), 711–719 (2016). 10.5435/JAAOS-D-15-00175 27579811

[41] Ricci, W. M. *et al.* Risk factors for failure of locked plate fixation of distal femur fractures: an analysis of 335 cases. *J. Orthop. Trauma* **28**(2), 83–89 (2014). 23760176 10.1097/BOT.0b013e31829e6dd0

[42] Lambert, K. L. The weight-bearing function of the fibula: a strain gauge study. *J. Bone Joint Surg. Am.* **53**, 507–513 (1971). 5580009 10.2106/00004623-197153030-00007

[43] Goh, J. C., Eng Hin, L. E., Eng Joo, A. N., Bayon, P. & Pho, R. W. Biomechanical study on the load-bearing characteristics of the fibula and the effects of fibular resection. *Clin. Orthop. Related Res.* **279**, 223–228 (1992). 10.1097/00003086-199206000-00028 1600659

[44] Raja Izaham, R. M., Abdul Kadir, M. R., Abdul Rashid, A. H., Hossain, M. G. & Kamarul, T. Finite element analysis of Puddu and Tomofix plate fixation for open wedge high tibial osteotomy. *Injury* **43**, 898–902 (2012). 22204773 10.1016/j.injury.2011.12.006

