# Author Correction: Comparison of tibial fracture plate length, placement, and fibular integrity effects on plate integrity through finite element analysis

**DOI:** 10.1038/s41598-024-69579-x

**Published:** 2024-08-12

**Authors:** Byung Hoon Lee, Yeokyung Kang, Sung Ha Cho, Myung Moon, Jae Ang Sim, Jungsung Kim

**Affiliations:** 1 Department of Orthopaedic Surgery, Gachon University College of Medicine, 21, Namdong-Daero 774 Beon-Gil, Namdong-Gu, Incheon, Republic of Korea; 2 Central Research and Development Center, Corentec Company Ltd., 33-2, Banpo-Daero 20-Gil, Seocho-Gu, Seoul, Republic of Korea; 3 Gachon University College of Medicine, Incheon, Republic of Korea

Correction to: *Scientific Reports* 10.1038/s41598-024-64990-w, published online 24 June 2024

The original version of this Article contained an error in the Funding section.

“This work was supported by the National Research Foundation of Korea (NRF) grant funded by the Korean government (MSIT) (No. 2021M3I2A1077405), and by the Gachon University research fund of 2021 (GCU-2021–07290001).”

now reads:

“This work was supported by the National Research Foundation of Korea (NRF) grant funded by the Korean government (MSIT) (No. 2021M3I2A1077405), and by the Gachon University research fund of 2021 (GCU-2021–07290001). This research was conducted with support from the Industrial Innovation Infrastructure Construction Project sponsored by the Ministry of Trade, Industry and Energy (P0025775).”

The original Article has been corrected.

