# Supplementary material for: Comparison of tibial fracture plate length, placement, and fibular integrity effects on plate integrity through finite element analysis
Source: Sci Rep. 2024 Jun 24;14:14538. doi: 10.1038/s41598-024-64990-w (PMC11196683; doi:10.1038/s41598-024-64990-w)
Supplement: Supplementary file 1 — Supplementary Information. [file 41598_2024_64990_MOESM1_ESM.docx]

**Supplementary Tables**

**Supplementary Table S1**. Variation of the analysis model

| Model no. | Type of plate | Medial plate length | Lateral plate length | PFO |
| --- | --- | --- | --- | --- |
| D1 | Dual plate | 150 | 200 | X (Non-PFO) |
| D2 | Dual plate | 200 | 250 | X (Non-PFO) |
| M1 | Medial plate | 150 | - | X (Non-PFO) |
| M2 | Medial plate | 200 |  | X (Non-PFO) |
| M3 | Medial plate | 250 | - | X (Non-PFO) |
| L1 | Lateral plate | - | 150 | X (Non-PFO) |
| L2 | Lateral plate | - | 200 | X (Non-PFO) |
| L3 | Lateral plate | - | 250 | X (Non-PFO) |
| D1P | Dual plate | 150 | 200 | O (PFO) |
| D2P | Dual plate | 200 | 250 | O (PFO) |
| M1P | Medial plate | 150 | - | O (PFO) |
| M2P | Medial plate | 200 |  | O (PFO) |
| M3P | Medial plate | 250 | - | O (PFO) |
| L1P | Lateral plate | - | 150 | O (PFO) |
| L2P | Lateral plate | - | 200 | O (PFO) |
| L3P | Lateral plate | - | 250 | O (PFO) |

PFO, proximal fibula osteotomy.

**Supplementary Table S2.** The number of nodes and elements in each model

| **Model no.** | **Number of nodes** | **Number of elements** |
| --- | --- | --- |
| **D1** | 984,997 | 617,253 |
| **D2** | 1,059,342 | 659,739 |
| **M1** | 811,102 | 521,614 |
| **M2** | 844,362 | 541,335 |
| **M3** | 888,893 | 566,898 |
| **L1** | 752,620 | 481,124 |
| **L2** | 814,275 | 520,710 |
| **L3** | 854,081 | 542,858 |
| **D1P** | 982,586 | 615,615 |
| **D2P** | 1,055,817 | 657,648 |
| **M1P** | 808,589 | 519,868 |
| **M2P** | 843,271 | 540,675 |
| **M3P** | 885,777 | 564,733 |
| **L1P** | 781,667 | 501,307 |
| **L2P** | 811,030 | 518,376 |
| **L3P** | 851,083 | 540,582 |

**Supplementary Table S3.** Material properties

|  | **Young’s Modulus (MPa)** | **Poisson’s ratio** | **Stiffness (N/mm, per 1 spring)** |
| --- | --- | --- | --- |
| **Cortical bone** ^[22]^ | E_x_=6,910 | V_xy_=0.49 | - |
|  | E_y_=8,510 | V_xz_=0.12 | - |
|  | E_z_=18,400 | V_yz_=0.14 | - |
| **Cancellous bone** ^[23]^ | 1,061 | 0.225 | - |
| **Cartilage** ^[24]^ | 12 | 0.45 | - |
| **Meniscus** ^[24]^ | 80 | 0.3 | - |
| **MCL** ^[25]^ | - | - | 24 |
| **LCL** ^[25]^ | - | - | 23.2 |
| **Proximal anterior tibiofibular ligament** ^[26]^ | - | - | 44.3 |
| **Proximal posterior tibiofibular ligament** ^[26]^ | - | - | 36.3 |
| **Distal anterior tibiofibular ligament** ^[27]^ | - | - | 26 |
| **Distal posterior tibiofibular ligament** ^[27]^ | - | - | 33.7 |
| **Anterior interosseous membrane** ^[21, 27]^ | - | - | 39 |
| **Posterior interosseous membrane** ^[21, 27]^ | - | - | 39 |

MCL, medial collateral ligament; LCL, lateral collateral ligament.

**Supplementary Table S4.** The contact conditions

| **Contact body** | **Contact type** |
| --- | --- |
| Cortical – Cancellous bone (Femur, Tibia, Fibula) | Bonded |
| Femur bone – Femur cartilage | Bonded |
| Tibia bone – Tibia cartilage | Bonded |
| Meniscus – Tibia cartilage | Bonded |
| Femur cartilage – Meniscus | Frictional, μ=0.2 |
| Femur cartilage – Tibia cartilage | Frictional, μ=0.2 |
| Tibia bone – Fibula bone | No-separation, μ=0 |
| Plate – Screw | Bonded |
| Screw – Bone | Bonded |

**Supplementary Table S5.** The von-Mises Stresses calculated for the tibia fracture plate model without PFO

| **Model No.** | **D1** | | **D2** | | **M1** | **M2** | **M3** | **L1** | **L2** | **L3** |
| --- | --- | --- | --- | --- | --- | --- | --- | --- | --- | --- |
| **Type of plate** | **Dual** | | **Dual** | | **Medial** | **Medial** | **Medial** | **Lateral** | **Lateral** | **Lateral** |
|  | **M**  **150** | **L**  **200** | **M**  **200** | **L**  **250** | **M**  **150** | **M**  **200** | **M**  **250** | **L**  **150** | **L**  **200** | **L**  **250** |
| **Peak plate stress (MPa)** | 154.64 | 75.14 | 145.63 | 87.55 | 256.71 | 241.46 | 181.66 | 1510.6 | 1531.4 | 1499.4 |
| **Average plate stress (MPa)** | 14.06 | 9.56 | 12.56 | 8.68 | 20.03 | 17.95 | 17.51 | 80.31 | 65.02 | 59.50 |
| **Anterior neck peak stress (MPa)** | 62.46 | 75.14 | 51.00 | 73.53 | 113.06 | 105.26 | 101.18 | 413.64 | 403.74 | 366.98 |
| **Posterior neck peak stress (MPa)** | 68.48 | 45.9 | 61.38 | 37.57 | 100.08 | 99.92 | 96.96 | 281.45 | 273.07 | 243.95 |

PFO, proximal fibula osteotomy.

**Supplementary Table S6.** Displacement of the tibia fracture plate model without PFO

| **Model No.** | **D1** | | **D2** | | **M1** | **M2** | **M3** | **L1** | **L2** | **L3** |
| --- | --- | --- | --- | --- | --- | --- | --- | --- | --- | --- |
| **Type of plate** | **Dual** | | **Dual** | | **Medial** | **Medial** | **Medial** | **Lateral** | **Lateral** | **Lateral** |
|  | **M**  **150** | **L**  **200** | **M**  **200** | **L**  **250** | **M**  **150** | **M**  **200** | **M**  **250** | **L**  **150** | **L**  **200** | **L**  **250** |
| **Peak displacement (mm)** | 1.16 | 1.13 | 0.808 | 0.782 | 1.46 | 1.25 | 1.02 | 3.75 | 3.82 | 3.96 |
| **Average displacement (mm)** | 0.682 | 0.536 | 0.392 | 0.298 | 0.822 | 0.585 | 0.376 | 1.204 | 0.976 | 0.801 |

PFO, proximal fibula osteotomy.

**Supplementary Table S7.** The von-Mises Stresses calculated for the tibia fracture plate model with PFO

| **Model No.** | **D1P** | | **D2P** | | **M1P** | **M2P** | **M3P** | **L1P** | **L2P** | **L3P** |
| --- | --- | --- | --- | --- | --- | --- | --- | --- | --- | --- |
| **Type of plate** | **Dual** | | **Dual** | | **Medial** | **Medial** | **Medial** | **Lateral** | **Lateral** | **Lateral** |
|  | **M**  **150** | **L**  **200** | **M**  **200** | **L**  **250** | **M**  **150** | **M**  **200** | **M**  **250** | **L**  **150** | **L**  **200** | **L**  **250** |
| **Peak plate stress (MPa)** | 227.19 | 173.37 | 257.8 | 145.6 | 675.39 | 781.34 | 538.48 | 2512.2 | 3274.6 | 3235.3 |
| **Average plate stress (MPa)** | 18.98 | 14.94 | 17.26 | 14.47 | 49.82 | 42.47 | 38.39 | 121.57 | 93.59 | 88.42 |
| **Anterior neck peak stress (MPa)** | 116.01 | 114.2 | 113.62 | 107.68 | 372.35 | 352.52 | 293.73 | 320.83 | 312.81 | 287.89 |
| **Posterior neck peak stress (MPa)** | 98.3 | 98.21 | 101.62 | 96.82 | 234.94 | 224.33 | 210.92 | 184.19 | 167.04 | 144.99 |

PFO, proximal fibula osteotomy.

**Supplementary Table S8.** Displacements calculated for the tibia fracture plate model with PFO

| **Model No.** | **D1P** | | **D2P** | | **M1P** | **M2P** | **M3P** | **L1P** | **L2P** | **L3P** |
| --- | --- | --- | --- | --- | --- | --- | --- | --- | --- | --- |
| **Type of plate** | **Dual** | | **Dual** | | **Medial** | **Medial** | **Medial** | **Lateral** | **Lateral** | **Lateral** |
|  | **M**  **150** | **L**  **200** | **M**  **200** | **L**  **250** | **M**  **150** | **M**  **200** | **M**  **250** | **L**  **150** | **L**  **200** | **L**  **250** |
| **Peak displacement (mm)** | 1.24 | 1.24 | 1.06 | 1.07 | 1.96 | 2.04 | 2.15 | 7.31 | 7.12 | 8.16 |
| **Average displacement (mm)** | 0.685 | 0.546 | 0.474 | 0.372 | 0.824 | 0.683 | 0.558 | 1.762 | 1.325 | 1.204 |

PFO, proximal fibula osteotomy.
